# Efficacy and Safety of Low‐Dose Edoxaban by Body Weight in Very Elderly Patients With Atrial Fibrillation: A Subanalysis of the Randomized ELDERCARE‐AF Trial

**DOI:** 10.1161/JAHA.123.031506

**Published:** 2024-01-19

**Authors:** Masaharu Akao, Takeshi Yamashita, Masayuki Fukuzawa, Takuya Hayashi, Ken Okumura

**Affiliations:** ^1^ Department of Cardiology National Hospital Organization Kyoto Medical Center Kyoto Japan; ^2^ The Cardiovascular Institute Tokyo Japan; ^3^ Cardiovascular Group, Primary Medical Science Department, Japan Business Unit, Daiichi Sankyo Co., Ltd. Tokyo Japan; ^4^ Data Intelligence Group, Data Intelligence Department, Digital Transformation Management Division, Daiichi Sankyo Co., Ltd. Tokyo Japan; ^5^ Division of Cardiology Saiseikai Kumamoto Hospital Kumamoto Japan

**Keywords:** atrial fibrillation, bleeding, body weight, low‐dose edoxaban, Arrhythmias

## Abstract

**Background:**

The ELDERCARE‐AF trial showed that low‐dose edoxaban benefits elderly patients with nonvalvular atrial fibrillation considered ineligible for standard oral anticoagulants due to high bleeding risk, but whether this applied to patients with extremely low body weight was unclear.

**Methods and Results:**

This was a prespecified subanalysis by body weight (≤45, >45 kg) of the phase 3, multicenter, randomized, double‐blind, placebo‐controlled, event‐driven ELDERCARE‐AF trial, which compared low‐dose edoxaban (15 mg once daily) with placebo in Japanese patients considered ineligible for oral anticoagulants at the recommended therapeutic strength or the approved doses. The primary efficacy and safety end points were stroke or systemic embolism and major bleeding (International Society on Thrombosis and Hemostasis definition), respectively. The ≤45‐kg weight group included 374/984 patients (38.0%), and the >45‐kg group included 610/984 patients (62.0%). The stroke or systemic embolism rate was lower with edoxaban than placebo in both weight groups (≤45 kg: hazard ratio [HR], 0.36 [95% CI, 0.16–0.80]; >45 kg: HR, 0.31 [95% CI, 0.13–0.73]; interaction *P*=0.82). Major bleeding incidence was numerically higher with edoxaban than placebo (≤45 kg: HR, 3.05 [95% CI, 0.84–11.11]; >45 kg: HR, 1.40 [95% CI, 0.56–3.48), with no interaction with body weight (interaction *P*=0.33). All‐cause mortality was higher in the ≤45‐kg group, with no significant difference between treatment groups.

**Conclusions:**

The benefit of edoxaban 15 mg was consistent in elderly patients with atrial fibrillation and extremely low body weight, though clinicians must remain vigilant about the risk of major bleeding, especially gastrointestinal bleeding.

**Registration Information:**

ClinicalTrials.gov. Identifier: NCT02801669.

**Nonstandard Abbreviations and Acronyms**
DOAC: direct oral anticoagulant
OAC: oral anticoagulant
SSE: stroke or systemic embolism

**Clinical Perspective**

**What Is New?**

- Among elderly patients with nonvalvular atrial fibrillation who were considered ineligible for standard oral anticoagulants due to their high bleeding risk, treatment with edoxaban (15 mg) reduced the incidence of stroke/systemic embolism compared with placebo in both body weight groups (≤45 and >45 kg).
- The incidence of major bleeding events (predominantly gastrointestinal bleeding) was numerically higher in the edoxaban group than in the placebo group, but there was no interaction with body weight.

**What Are the Clinical Implications?**

- The benefit of edoxaban 15 mg was consistent in elderly patients with atrial fibrillation and a very low body weight; however, clinicians must remain vigilant about the risk of major bleeding, particularly gastrointestinal bleeding.

Atrial fibrillation (AF), which is the most common cardiac arrhythmia, has ischemic stroke as its most severe complication, leading to increased morbidity and mortality of affected patients.^1^ To prevent stroke, clinical guidelines recommend the use of oral anticoagulants (OACs), but the perceived risk of bleeding is a major obstacle to prescribing OACs.^2^ Treatment with OACs can lead to bleeding adverse events, and they are particularly difficult to use in patients at high risk for bleeding; elderly status, low body weight, and renal impairment are known risk factors for bleeding with OACs.^3^

In patients at such a high risk of bleeding who are ineligible for OAC treatment, the ELDERCARE‐AF (Edoxaban Low‐Dose for Elder Care Atrial Fibrillation Patients) trial, which was a randomized, placebo‐controlled study comparing low‐dose edoxaban (15 mg once daily) with placebo, showed a significant reduction of stroke or systemic embolism (SSE) with edoxaban 15 mg compared with placebo (hazard ratio [HR], 0.34 [95% CI, 0.19–0.61]), without a significant increase in major bleeding (HR, 1.87 [95% CI, 0.90–3.89]).^4^ In addition, subgroup analyses of ELDERCARE‐AF confirmed the consistent benefit of edoxaban 15 mg irrespective of age (80–84 years, 85–89 years, and ≥90 years) and renal function (creatinine clearance 15–<30 mL/min, 30–50 mL/min, and >50 mL/min).^5^, ^6^

With respect to low body weight, previous studies have reported the effectiveness and safety of direct OACs (DOACs) for preventing stroke in patients with AF and low body weight. The ENGAGE AF‐TIMI 48 (Effective Anticoagulation with Factor Xa Next Generation in Atrial Fibrillation‐Thrombolysis in Myocardial Infarction 48) trial reported similar efficacy of the DOACs regimen at the recommended dose across 3 body weight groups, including the low body weight group defined as <55 kg.^7^ However, the efficacy and safety of OACs in patients with AF who are very underweight (≤45 kg) have yet to be demonstrated. This is an important issue, because relatively small and elderly patients weighing <45 kg are not uncommon in Japan and other Asian countries.

This subanalysis was conducted to evaluate the efficacy and safety of edoxaban in patients with AF with extremely low body weight. We used data from the ELDERCARE‐AF trial, which compared low‐dose edoxaban with placebo in Japanese patients aged ≥80 years with AF, and assessed clinical outcomes according to body weight (≤45 or >45 kg).

## Methods

### Data Availability

The data sets used in the current analysis are available from the corresponding author upon reasonable request.

### Study Design

The present study was a prespecified subanalysis of data from the ELDERCARE‐AF trial, a phase 3, multicenter, randomized, double‐blind, placebo‐controlled, event‐driven, superiority trial. The trial design and main outcomes have been previously described.^4^, ^8^ In brief, ELDERCARE‐AF was conducted at 164 centers in Japan.^4^ Following a screening period (up to 30 days), eligible patients were assigned in a 1:1 ratio to treatment with either edoxaban (15 mg once daily) or matching placebo until study completion (based on occurrence of SSE events).^8^ An interactive response technology system was used for randomization, per a schedule prepared by an independent biostatistician, and all other participating individuals (ie, patients, investigators, and sponsor) were blinded to group assignments.^4^

The ELDERCARE‐AF trial was conducted in accordance with the ethical principles outlined in the Declaration of Helsinki, and the institutional review board of each participating site approved the protocol. All participants provided their written, informed consent. The trial was registered at ClinicalTrials.gov (NCT02801669). The present study was conducted in accordance with the Consolidated Standards of Reporting Trials (CONSORT) guidelines.

### Patients Edoxaban Low‐Dose for Elder Care Atrial Fibrillation Patients

The eligibility criteria for the ELDERCARE‐AF trial included the following: age ≥80 years; CHADS_2_ risk score (congestive heart failure, hypertension, age ≥75 years, diabetes, and history of stroke) ≥2.^4^, ^8^ All patients were of Japanese ethnicity and had to be considered ineligible for OACs such as warfarin, apixaban, dabigatran, edoxaban, or rivaroxaban at the recommended therapeutic strength or the approved doses due to at least 1 of the following reasons: low creatinine clearance (15–30 mL/min); history of bleeding from a critical area/organ or gastrointestinal bleeding; low body weight (≤45 kg); ongoing use of nonsteroidal anti‐inflammatory drugs; and current use of an antiplatelet medication. Excluded from the study were patients with moderate–severe mitral stenosis and/or mechanical heart valves.

For this subanalysis, 45 kg was used as a cutoff to stratify patients into 2 body weight groups (ie, ≤45 and >45 kg). This cutoff was defined based on the design of the main ELDERCARE‐AF trial,^4^, ^8^ in which a body weight of ≤45 kg was specified as one of the ineligibility criteria for OAC therapy at the recommended therapeutic strength or the approved doses. As a result of this enrollment criterion, many patients included in the overall ELDERCARE‐AF population had a body weight of 40 to 45 kg, thereby ensuring sufficient patient numbers in each body weight subgroup for comparative analysis. In addition, an exploratory analysis was performed with all patients divided into 4 subgroups based on quartiles of body weight: >57.9 kg, >49.0 to ≤57.9 kg, >42.0 to ≤49.0 kg, and ≤42.0 kg.

### End Points

The primary efficacy end point was SSE. The secondary efficacy end point was the net clinical outcome and all‐cause mortality, with the net clinical outcome defined as the composite of stroke, systemic embolism, major bleeding, and all‐cause mortality. The primary safety end point was major bleeding as defined by the International Society on Thrombosis and Hemostasis. The secondary safety end point was major bleeding and clinically significant nonmajor bleeding. Full definitions and details of how each end point was determined have been described.^4^ Other secondary efficacy and safety end points have also been previously defined.^4^

### Statistical Analysis

The ELDERCARE‐AF study was event‐driven, with the sample size selected to show superiority of edoxaban over placebo for the prevention of SSE.^4^, ^8^ For this prespecified subanalysis, no additional sample size calculations were conducted, and all data were obtained from the overall trial population. The primary efficacy analysis was conducted in the intention‐to‐treat population, and the safety analysis was conducted in the safety analysis set (all patients who received at least 1 dose of the trial drug).

For this prespecified subanalysis, to evaluate patients based on body weight at baseline, patients included in the ELDERCARE‐AF trial were further classified into 2 groups by body weight (≤45 and >45 kg). Their demographic and clinical characteristics are described using distributions and summary statistics, with continuous variables reported as mean and SD values.

The time to first SSE event was analyzed using a Cox proportional hazards model by treatment group, with the CHADS_2_ score (2 or ≥3 points) as a covariate and a 2‐sided significance level of 5%. Relative risks were estimated using HRs with 95% CIs. The secondary efficacy end points were analyzed using the same method. The Kaplan**–**Meier method was used to estimate the cumulative incidence of efficacy events by treatment group. Bleeding events were summarized by group and analyzed during the on‐treatment period, including the treatment period and up to 3 days after the last dose of the study drug or the end of the study.

A Cox proportional hazards model was used to evaluate the effects of body weight as a categorical variable on efficacy and safety outcomes. SAS software, version 9.4 (SAS Institute, Cary, NC) was used to perform the statistical analyses.

## Results

### Patients

**Figure 1:**
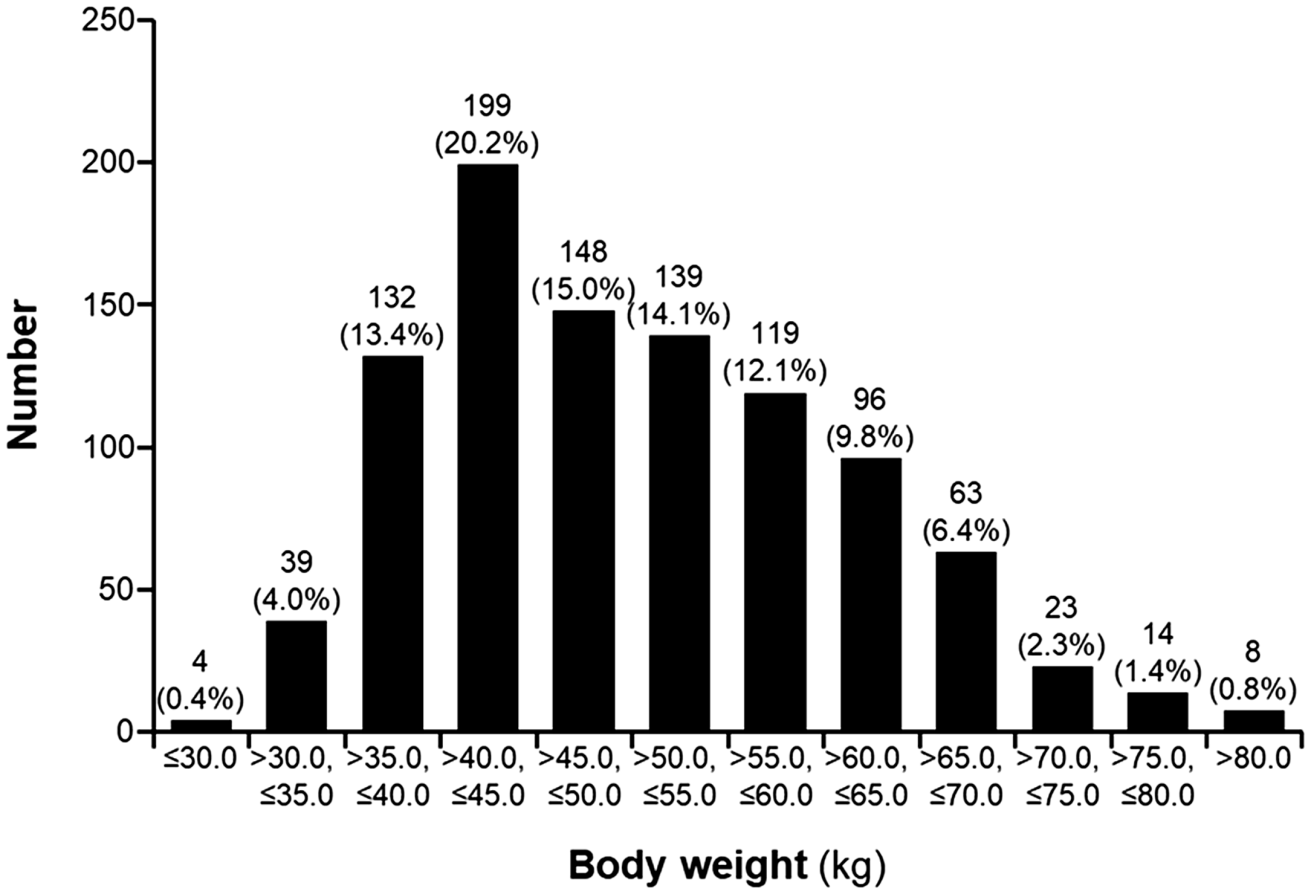
Distribution of participants by body weight.

A total of 1086 patients were enrolled, with 984 randomly assigned to either the edoxaban group (492 patients) or the placebo group (492 patients; Figure S1). The participants were then further divided into 2 subgroups by body weight (374 patients [38.0%] in the ≤45‐kg group, 610 [62.0%] in the >45‐kg group). Most of the patients in the ≤45‐kg group weighed 40 to 45 kg (199/374 [53.2%]; Figure 1).

Table 1 shows the patients' baseline characteristics by body weight subgroup. The mean body weight (±SD) for each group was 39.8±3.9 kg and 57.2±8.4 kg in the ≤45‐kg group and >45‐kg group, respectively. The mean age (±SD) for each group was 87.8±4.4 years and 85.8±4.0 years for the ≤45‐kg group and >45‐kg group, respectively. The proportion of male patients was higher in the >45‐kg group. The body mass index (BMI), creatinine clearance, prevalence of comorbidities (coronary artery disease, dyslipidemia, and diabetes), HAS‐BLED score, continuous use of nonsteroidal anti‐inflammatory drugs, and use of an antiplatelet drug all showed decreases with lower body weight. Table 2 shows the patients' baseline characteristics in the edoxaban and placebo groups by body weight subgroup (≤45 and >45 kg). The characteristics of each weight subgroup did not differ between the edoxaban and placebo groups.

**Table 1:** Patients' Baseline Characteristics by Body Weight Subgroup

Characteristic	≤45 kg	>45 kg
	(N=374)	(N=610)
Age, y	87.8±4.4	85.8±4.0
Male sex	48 (12.8)	371 (60.8)
Paroxysmal atrial fibrillation	191 (51.1)	272 (44.6)
Weight, kg	39.8±3.9	57.2±8.4
BMI, kg/m^2^ *	19.3±2.3	23.9±3.3
Creatinine clearance, mL/min		
Mean	31.2 ± 11.4	39.4±15.1
<30	204 (54.5)	197 (32.3)
≥30	170 (45.5)	413 (67.7)
Coronary artery disease	61 (16.3)	196 (32.1)
Dementia	95 (25.4)	65 (10.7)
Dyslipidemia	129 (34.5)	321 (52.6)
History of falling within past year	150 (40.1)	190 (31.1)
CHADS_2_ score†		
Mean	3.0±1.0	3.1±1.2
≤2	141 (37.7)	222 (36.4)
≥3	233 (62.3)	388 (63.6)
Risk factor for thromboembolism		
Congestive heart failure	227 (60.7)	306 (50.2)
Hypertension	290 (77.5)	520 (85.2)
Age ≥75 y	374 (100.0)	610 (100.0)
Diabetes	55 (14.7)	170 (27.9)
Previous stroke or TIA	84 (22.5)	152 (24.9)
CHA_2_DS_2_‐VASc score‡	5.0±1.2	4.8±1.3
HAS‐BLED score§	2.0±0.9	2.5±0.8
Reason for OAC ineligibility		
Severe renal impairment (creatinine clearance <30 mL/min)	204 (54.5)	199 (32.6)
History of bleeding from critical area or organ	53 (14.2)	169 (27.7)
Intracranial	21 (5.6)	59 (9.7)
Gastrointestinal	29 (7.8)	98 (16.1)
Intraocular	2 (0.5)	5 (0.8)
Other||	1 (0.3)	13 (2.1)
Continuous use of NSAIDs	77 (20.6)	240 (39.3)
Use of an antiplatelet drug	134 (35.8)	395 (64.8)
Aspirin	71 (19.0)	220 (36.1)
Clopidogrel	37 (9.9)	97 (15.9)
Other#	27 (7.2)	80 (13.1)
Frailty category**		
Robust	6 (1.6)	55 (9.0)
Prefrail	157 (42.0)	324 (53.1)
Frail	191 (51.1)	211 (34.6)
Could not be evaluated	9 (2.4)	8 (1.3)
Missing data	11 (2.9)	12 (2.0)
History of oral anticoagulant therapy		
Yes	142 (38.0)	281 (46.1)
Warfarin	77 (20.6)	166 (27.2)
Direct oral anticoagulant	95 (25.4)	156 (25.6)
Unknown	1 (0.3)	0 (0.0)
No	232 (62.0)	329 (53.9)

Data are shown as n (%) or mean±SD values.

BMI indicates body mass index; NSAIDs, nonsteroidal anti‐inflammatory drugs; OAC, oral anticoagulant; and TIA, transient ischemic attack.

* Data were missing for 2 patients in the ≤45‐kg group and 1 patient in the >45‐kg group.

^†^ CHADS_2_ scores ranged from 0–6 points, with higher scores indicating a greater risk of stroke. Previous stroke or TIA was assigned 2 points, and congestive heart failure, hypertension, diabetes, and age ≥75 y were each assigned 1 point.

^‡^ CHA_2_DS_2_‐VASc scores ranged from 0–9 points, with higher scores indicating a greater risk of stroke. Previous stroke or TIA and age ≥75 years were each assigned 2 points, and congestive heart failure, hypertension, diabetes, age 65–74 y, female sex, and history of vascular disease were each assigned 1 point.

^§^ HAS‐BLED scores ranged from 0–9 points, with higher scores indicating a greater risk of bleeding. Abnormal kidney function and abnormal liver function were assigned 1 point each; use of antiplatelets, use of NSAIDs, and use of alcohol concomitantly were assigned 1 point each (for a total of 1 or 2 points), and hypertension, stroke, history of bleeding or a predisposition to bleeding, labile international normalized ratio, and older age (>65 y) were each assigned 1 point.

^||^ Other category includes bladder, hemoperitoneum, intra‐articular in the right knee, left retroperitoneal hematoma, lung, pericardial space, pulmonary alveolar hemorrhage, right iliopsoas, and shoulder joint.

^#^ Other includes beraprost, cilostazol, dilazep, dipyridamole, ethyl icosapentate, limaprost alfadex, prasugrel, sarpogrelate, and ticlopidine.

** Frailty was assessed using 5 measures of physical condition; a score of 0 indicated robust, a score of 1 or 2 indicated prefrail, and a score of ≥3 indicated frail. Data were available for 354 patients in the ≤45‐kg group and 590 patients in the >45‐kg group.

**Table 2:** Patients' Baseline Characteristics in the Edoxaban and Placebo Groups by Body Weight Subgroup

Characteristic	Edoxaban 15 mg		Placebo	
	≤45 kg (N=188)	>45 kg (N=304)	≤45 kg (N=186)	>45 kg (N=306)
Age, y	87.6±4.4	86.1±4.0	87.9±4.5	85.6±3.9
Male sex	21 (11.2)	191 (62.8)	27 (14.5)	180 (58.8)
Paroxysmal atrial fibrillation	98 (52.1)	139 (45.7)	93 (50.0)	133 (43.5)
Weight, kg	39.7±3.9	57.3±7.9	39.9±3.8	57.0±8.9
BMI, kg/m^2^ *	19.3±2.3	23.8±3.1	19.3±2.3	24.0±3.5
Creatinine clearance, mL/min				
Mean	31.6±10.7	39.3±15.4	30.8±12.1	39.6±14.9
<30	96 (51.1)	101 (33.2)	108 (58.1)	96 (31.4)
≥30	92 (48.9)	203 (66.8)	78 (41.9)	210 (68.6)
Coronary artery disease	30 (16.0)	100 (32.9)	31 (16.7)	96 (31.4)
Dementia	39 (20.7)	31 (10.2)	56 (30.1)	34 (11.1)
Dyslipidemia	71 (37.8)	155 (51.0)	58 (31.2)	166 (54.2)
History of falling within past year	63 (33.5)	91 (29.9)	87 (46.8)	99 (32.4)
CHADS_2_ score†				
Mean	2.9±1.0	3.1±1.1	3.0±1.1	3.2±1.2
≤2	70 (37.2)	111 (36.5)	71 (38.2)	111 (36.3)
≥3	118 (62.8)	193 (63.5)	115 (61.8)	195 (63.7)
Risk factor for thromboembolism				
Congestive heart failure	115 (61.2)	144 (47.4)	112 (60.2)	162 (52.9)
Hypertension	152 (80.9)	260 (85.5)	138 (74.2)	260 (85.0)
Age ≥75 y	188 (100.0)	304 (100.0)	186 (100.0)	306 (100.0)
Diabetes	25 (13.3)	90 (29.6)	30 (16.1)	80 (26.1)
Previous stroke or TIA	37 (19.7)	73 (24.0)	47 (25.3)	79 (25.8)
CHA_2_DS_2_‐VASc score‡	5.0±1.2	4.8±1.3	5.0±1.2	4.9±1.3
HAS‐BLED score§	2.0±0.9	2.4±0.8	2.1±0.9	2.5±0.9
Reasons for OAC ineligibility				
Severe renal impairment (creatinine clearance <30 mL/min)	96 (51.1)	102 (33.6)	108 (58.1)	97 (31.7)
History of bleeding from critical area or organ	30 (16.0)	80 (26.3)	23 (12.4)	89 (29.1)
Intracranial	12 (6.4)	29 (9.5)	9 (4.8)	30 (9.8)
Gastrointestinal	16 (8.5)	45 (14.8)	13 (7.0)	53 (17.3)
Intraocular	1 (0.5)	2 (0.7)	1 (0.5)	3 (1.0)
Other ||	1 (0.5)	5 (1.6)	0 (0.0)	8 (2.6)
Low body weight (≤45 kg)	188 (100.0)	0 (0.0)	186 (100.0)	0 (0.0)
Continuous use of NSAIDs	41 (21.8)	108 (35.5)	36 (19.4)	132 (43.1)
Use of an antiplatelet drug	64 (34.0)	196 (64.5)	70 (37.6)	199 (65.0)
Aspirin	36 (19.1)	98 (32.2)	35 (18.8)	122 (39.9)
Clopidogrel	16 (8.5)	55 (18.1)	21 (11.3)	42 (13.7)
Other #	12 (6.4)	44 (14.5)	15 (8.1)	36 (11.8)
Frailty category**				
Robust	4 (2.1)	28 (9.2)	2 (1.1)	27 (8.8)
Prefrail	90 (47.9)	167 (54.9)	67 (36.0)	157 (51.3)
Frail	85 (45.2)	100 (32.9)	106 (57.0)	111 (36.3)
Could not be evaluated	3 (1.6)	4 (1.3)	6 (3.2)	4 (1.3)
Missing data	6 (3.2)	5 (1.6)	5 (2.7)	7 (2.3)
History of oral anticoagulant therapy				
Yes	76 (40.4)	131 (43.1)	66 (35.5)	150 (49.0)
Warfarin	36 (19.1)	79 (26.0)	41 (22.0)	87 (28.4)
Direct oral anticoagulants	53 (28.2)	71 (23.4)	42 (22.6)	85 (27.8)
Unknown	1 (0.5)	0 (0.0)	0 (0.0)	0 (0.0)
No	112 (59.6)	173 (56.9)	120 (64.5)	156 (51.0)

Data are shown as n (%) or mean±SD values.

BMI indicates body mass index; NSAIDs, nonsteroidal anti‐inflammatory drugs; OAC, oral anticoagulant; and TIA, transient ischemic attack.

* Data were missing for 1 patient in the >45‐kg group with edoxaban and 2 patients in the ≤45‐kg group with placebo.

^†^ CHADS_2_ scores ranged from 0–6 points, with higher scores indicating a greater risk of stroke. Previous stroke or TIA was assigned 2 points, and congestive heart failure, hypertension, diabetes, and age ≥75 y were each assigned 1 point.

^‡^ CHA_2_DS_2_‐VASc scores ranged from 0–9 points, with higher scores indicating a greater risk of stroke. Previous stroke or TIA and age ≥75 years were each assigned 2 points, and congestive heart failure, hypertension, diabetes, age 65–74 y, female sex, and history of vascular disease were each assigned 1 point.

^§^ HAS‐BLED scores ranged from 0–9 points, with higher scores indicating a greater risk of bleeding. Abnormal kidney function and abnormal liver function were assigned 1 point each; use of antiplatelets, use of NSAIDs, and use of alcohol concomitantly were assigned 1 point each (for a total of 1 or 2 points), and hypertension, stroke, history of bleeding or a predisposition to bleeding, labile international normalized ratio, and older age (>65 y) were each assigned 1 point.

^||^ Other category includes bladder, hemoperitoneum, intra‐articular in the right knee, left retroperitoneal hematoma, lung, pericardial space, pulmonary alveolar hemorrhage, right iliopsoas, and shoulder joint.

^#^ Other includes beraprost, cilostazol, dilazep, dipyridamole, ethyl icosapentate, limaprost alfadex, prasugrel, sarpogrelate, and ticlopidine.

** Frailty was assessed using 5 measures of physical condition; a score of 0 indicated robust, a score of 1 or 2 indicated prefrail, and a score of ≥3 indicated frail. Data were available for 179, 295, 175, and 295 patients in the ≤45‐kg group with edoxaban, >45‐kg group with edoxaban, ≤45‐kg group with placebo, and >45‐kg group with placebo, respectively.

### Efficacy End Points by Body Weight Subgroup

**Figure 2:**
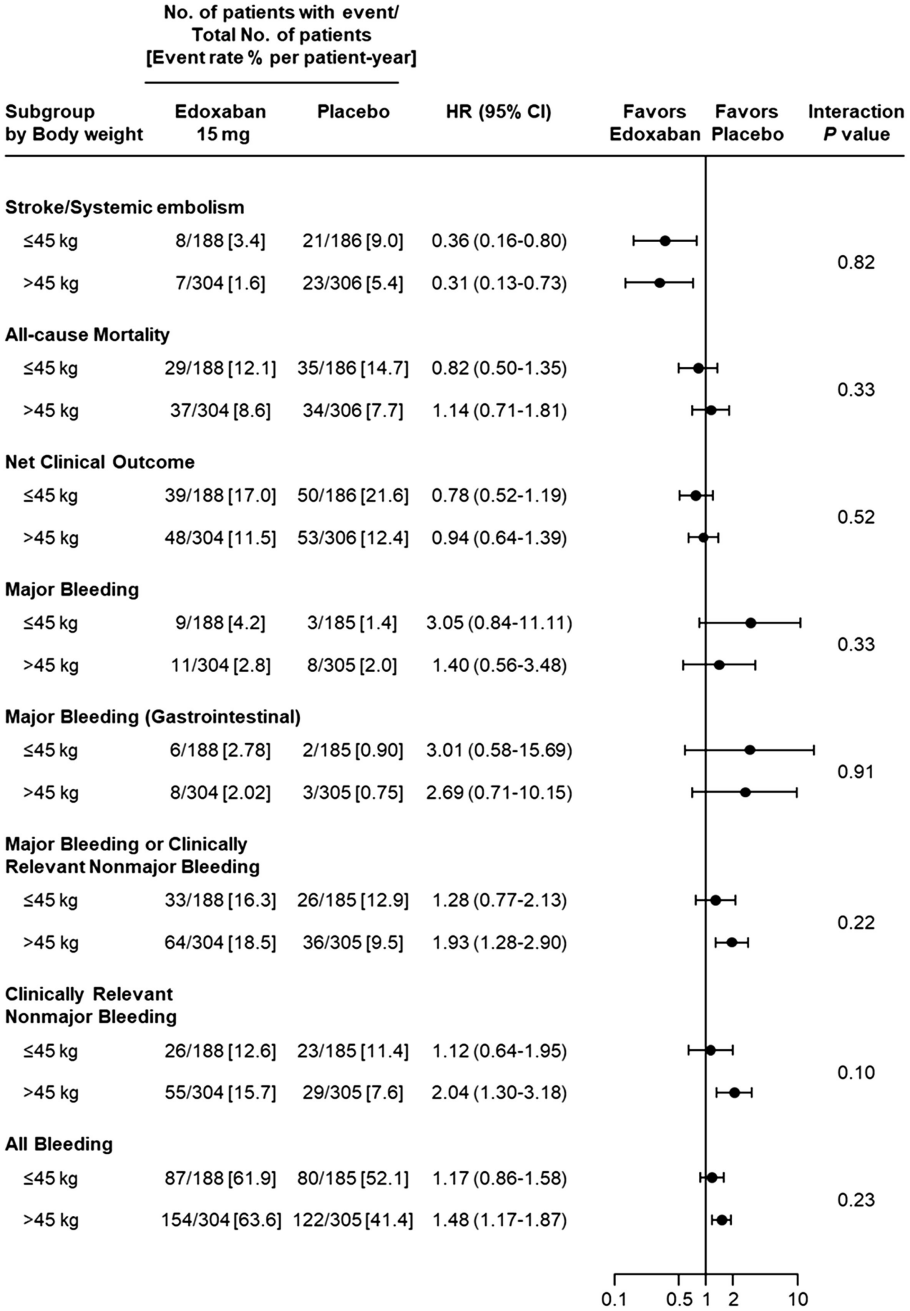
Effects of edoxaban on major efficacy and safety end points by body weight subgroup. HR indicates hazard ratio.

**Figure 3:**
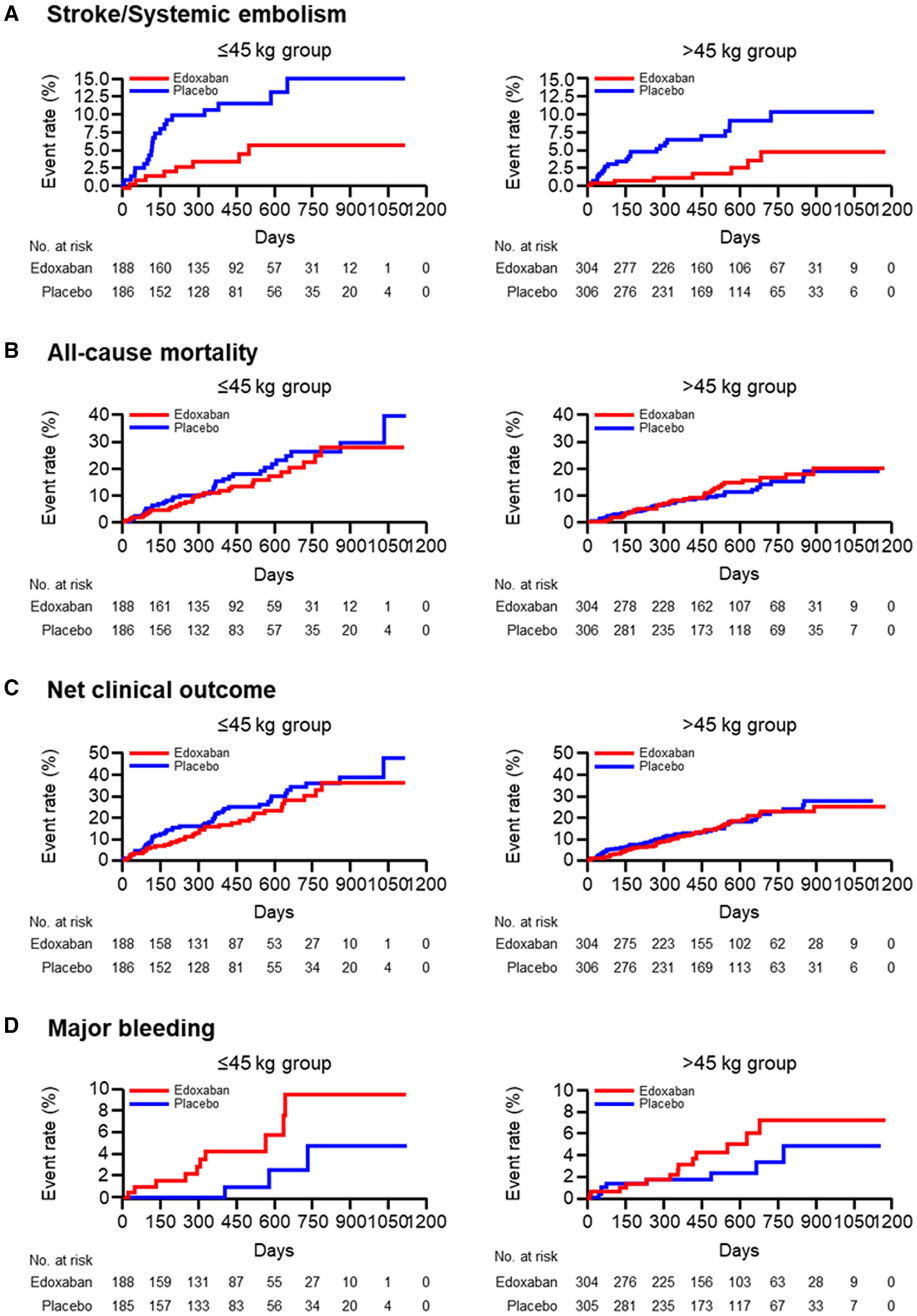
Kaplan–Meier curves for stroke/systemic embolism (A), all‐cause mortality (B), net clinical outcome (C), and major bleeding by body weight subgroup (D).

SSE, all‐cause mortality, and net clinical outcome were significantly higher in the ≤45‐kg group than in the >45‐kg group (Table 3). The incidences of efficacy end points in the edoxaban and placebo groups are shown in Figures 2 and 3. In the ≤45‐kg group, SSE occurred in 8 of 188 patients (3.4% per patient‐year) and 21 of 186 patients (9.0% per patient‐year) in the edoxaban group and placebo group, respectively (HR, 0.36 [95% CI, 0.16–0.80]). In the >45‐kg group, SSE occurred in 7 of 304 patients (1.6% per patient‐year) and 23 of 306 patients (5.4% per patient‐year) in the edoxaban group and placebo group, respectively (HR, 0.31 [95% CI, 0.13–0.73]). The incidence of SSE was significantly lower in the edoxaban group than in the placebo group, irrespective of body weight, and there was no interaction with body weight (*P*=0.82; Figures 2 and 3A). There were no significant differences between the edoxaban and placebo groups and no interaction with body weight for all‐cause mortality (*P*=0.33; Figures 2 and 3B) or net clinical outcome (*P*=0.52; Figures 2 and 3C).

**Table 3:** Efficacy and Safety End Points by Body Weight Subgroup

End point*	No. of patients with event/total no. of patients [event rate % per patient‐y]		Hazard ratio (95% CI)†
	≤45 kg	>45 kg	
Efficacy end point	N=374	N=610	
Stroke/systemic embolism	29/374 [6.2]	30/610 [3.5]	1.71 (1.02–2.84)
All‐cause mortality	64/374 [13.4]	71/610 [8.2]	1.64 (1.17–2.29)
Net clinical outcome	89/374 [19.3]	101/610 [12.0]	1.60 (1.20–2.13)
Safety end point	N=373	N=609	
Major bleeding	12/373 [2.8]	19/609 [2.4]	1.15 (0.56–2.38)
Major bleeding (gastrointestinal)	8/373 [1.8]	11/609 [1.4]	1.32 (0.53–3.31)
Major bleeding or clinically relevant nonmajor bleeding	59/373 [14.6]	100/609 [13.8]	1.05 (0.76–1.45)
Clinically relevant nonmajor bleeding	49/373 [12.0]	84/609 [11.5]	1.03 (0.72–1.46)
All bleeding	167/373 [56.8]	276/609 [51.4]	1.08 (0.89–1.31)

* The date of the last patient follow‐up was December 27, 2019, and the median duration of participation in the trial was 466.0 days (interquartile range, 293.5–708.0 days).

^†^ Indicates the probability of an event occurring in the ≤45‐kg weight group compared with the >45‐kg group. A hazard ratio of 1 indicates equal event probabilities in both groups; numbers >1 indicate a higher event probability in the ≤45‐kg weight group.

### Safety End Points by Body Weight Subgroup

There was no significant difference in any safety end points between the ≤45‐kg group and the >45‐kg group (Table 3). The incidences of safety end points in the edoxaban and placebo groups are shown in Figures 2 and 3. In the ≤45‐kg group, major bleeding occurred in 9 of 188 patients (4.2% per patient‐year) and 3 of 185 patients (1.4% per patient‐year) in the edoxaban group and placebo group, respectively; though the rate of major bleeding was numerically higher in the edoxaban group, it was not significant (HR, 3.05 [95% CI, 0.84–11.11]). In addition, in the >45‐kg group, major bleeding occurred in 11 of 304 patients (2.8% per patient‐year) and 8 of 305 patients (2.0% per patient‐year) in the edoxaban group and placebo group, respectively (Figures 2 and 3D). The most common major bleeding event was gastrointestinal bleeding in the edoxaban group and placebo group (n=6/9 and n=2/3 for the ≤45‐kg group; n=8/11 and n=3/8 for the >45‐kg group, respectively). In the ≤45‐kg patient group, the incidence of gastrointestinal bleeding was 2.8% per patient‐year (6/188) in the edoxaban group and 0.9% per patient‐year (2/185) in the placebo group. In addition, in the ≤45‐kg group, there were no cases of intracranial hemorrhage in either the edoxaban or the placebo groups. In the >45‐kg group, there were significant differences in major bleeding or clinically relevant nonmajor bleeding between the edoxaban and placebo groups (HR, 1.93 [95% CI, 1.28–2.90]), with no interaction with body weight (*P*=0.22), but no significant differences were observed in the ≤45‐kg group (HR, 1.28 [95% CI, 0.77–2.13]; Figure 2).

### Exploratory Analysis

In the exploratory analysis in the 4 body weight subgroups categorized by quartile (>57.9 kg; >49.0 to ≤57.9 kg; >42.0 to ≤49.0 kg; and ≤42.0 kg), SSE tended to be less frequent in the edoxaban group than in the placebo group in all subgroups. The net clinical outcome and all‐cause death were similar between the edoxaban and placebo groups in all subgroups, and major bleeding tended to be more frequent in the edoxaban 15‐mg group. However, there was no interaction with body weight in the incidences of these outcomes (Figure S2).

## Discussion

The present subanalysis of the ELDERCARE‐AF trial showed that, in elderly patients with AF aged ≥80 years, (1) the incidences of SSE, all‐cause death, and net clinical outcome were significantly higher in the ≤45 kg group than in the >45 kg group, whereas those of bleeding were comparable between the 2 groups; (2) treatment with edoxaban 15 mg consistently decreased the incidence of SSE compared with placebo in both body weight groups (≤45 and >45 kg); (3) the incidence of major bleeding events (predominantly gastrointestinal bleeding) was numerically higher in the edoxaban group than in the placebo group, but there were no interactions with body weight; and (4) there were no significant differences in the incidences of all‐cause death or the net clinical outcome between the edoxaban and placebo groups.

It is important to consider the present results in the light of previous studies.^9^, ^10^, ^11^, ^12^, ^13^ A systematic review of the efficacy of DOACs in preventing SSE in patients with AF or acute venous thromboembolism showed that there was an increased risk of thromboembolism in those with low body weight, defined as ≤60 kg, compared with those whose body weight was not low, with comparable bleeding outcomes across all body weights.^13^ Furthermore, a previous study reported that DOACs are more effective and safer than warfarin in patients with AF who were defined as underweight; in fact, use of DOACs was associated with a higher net clinical benefit than warfarin in patients who weighed <50 kg (HR for composite outcome, 0.665 [95% CI, 0.581–0.762]).^12^

In the ENGAGE AF‐TIMI 48 trial, 3 groups (warfarin, low‐dose edoxaban, and high‐dose edoxaban groups) were further classified by weight into low (≤55 kg), middle (79.8–84 kg), and high (≥120 kg) weight groups.^7^ In that study, patients with low body weight had higher incidences of SSE than those with middle and high body weights. Specifically, the rates of SSE for low body weight, middle body weight, and high body weight were 7.0%, 5.1%, and 2.9%, respectively (*P* trend <0.001). In addition, the rates of major bleeding were lower for patients with low body weight (2.7%) than for those with middle body weight (5.4%) and high body weight (5.0%). In the present study, 40 to 45 kg was the most common body weight range (Figure 1). Because 45 kg is the cutoff value for OAC therapy in the trial, this was the threshold used to stratify patients into 2 groups (≤45 kg and >45 kg). In the present study, patients with a lower body weight than in the ENGAGE AF‐TIMI 48 trial were included, specifically, those weighing <45 kg, and no interaction between weight and outcome was identified. The efficacy of edoxaban was similar in the ≤45 kg group, but caution should be exercised in clinical practice because major bleeding may occur more frequently: the annual incidences of major bleeding in the ≤45 kg and >45 kg groups were 4.2% and 2.8%, respectively, in the edoxaban group, and 1.4% and 2.0%, respectively, in the placebo group. Thus, the results of the present study may provide support for the efficacy of low‐dose edoxaban in patients with lower body weight.

In the Fushimi AF registry,^11^ a community‐based registry of patients with AF in Japan, the cutoff for low body weight was 50 kg, compared with 45 kg in the present study. In addition, our study included very elderly patients with bleeding risk, whereas the Fushimi AF registry was an all‐comer registry with no age or bleeding risk restrictions. Thus, the Fushimi AF registry had a wider range of patients than the ELDERCARE‐AF trial. Although the comparison should be made with caution, the results for the present placebo group were nevertheless similar to those from the Fushimi AF registry, which reported that the incidence of SSE was higher in the low body weight group (HR, 2.19 [95% CI, 1.57–3.04]; *P*<0.01), and major bleeding was similar between the body weight groups (HR, 1.05 [95% CI, 0.64–1.68]; *P*=0.84). The J‐ELD AF Registry, which enrolled Japanese patients aged 75 years and older receiving on‐label doses of apixaban, evaluated 3 groups of patients weighing >60 kg, between 50 and 60 kg, and <50 kg.^9^ The incidences of SSE were 1.69%, 1.82%, and 1.23% per year, respectively, whereas the all‐cause mortality rates were 2.02%, 2.67%, and 4.92% per year, respectively. On multivariate analysis, body weight <50 kg was not a significant independent risk factor for SSE or all‐cause mortality. The J‐RISK AF study, a pooled analysis of 5 major AF registries in Japan, classified patients with AF based on their BMI into 4 categories: underweight (BMI <18.5 kg/m^2^), normal (18.5–24.9 kg/m^2^), overweight (25.0–29.9 kg/m^2^), and obese (≥30 kg/m^2^).^10^ On Cox proportional hazards model analysis, underweight (BMI <18.5 kg/m^2^) was found to be a significant risk factor for ischemic stroke (HR, 1.55 [95% CI, 1.05–2.29]; *P*=0.03). However, the results of the present study cannot be directly compared with those of the J‐RISK AF study because weight was used instead of BMI to classify patients.

The present study suggests that edoxaban 15 mg could be a treatment option even in very underweight, elderly patients with AF, although the incidence of major bleeding with treatment was notable. However, caution should be exercised in this setting because major bleeding tended to increase in the underweight group. In fact, 6 of 9 major bleeding events in the ≤45 kg group were gastrointestinal bleeds, which is consistent with previous reports that DOACs increase gastrointestinal bleeding.^14^, ^15^, ^16^, ^17^

### Study Limitations

The results of the present study must be considered in light of some limitations. First, a substantial number of patients discontinued the trial because of their high‐risk backgrounds, but no patients were lost to follow‐up, and only 6 withdrew consent because of bleeding‐related concerns. Of the patients who did withdraw, most did so due to adverse events that were unrelated to bleeding or participation was no longer possible. Second, the present trial involved Japanese patients with AF; therefore, the results may not be applicable to other populations with AF. Finally, the sample size of the present trial was not powered for any of the subgroup analyses, resulting in relatively low event rates that caused some subgroup analyses to have very few events; therefore, the statistical power of the subgroup analyses was limited. The results of the subgroup analyses should be interpreted with caution because there may be unaccounted confounding factors.

## Conclusions

The results of the present study, which compared the placebo group and the edoxaban 15‐mg groups in ELDERCARE‐AF stratified by body weight (≤45 and >45 kg), suggest that edoxaban 15 mg may be considered in elderly high‐risk patients with AF with low body weight, while remaining vigilant about the risk of major bleeding, especially gastrointestinal bleeding.

## Sources of Funding

This trial was supported by Daiichi Sankyo Co., Ltd.

## Disclosures

Dr Akao reported receiving grants from Daiichi Sankyo Co., Ltd. Research; a commission fee for study design from Daiichi Sankyo Co., Ltd.; lecture fees from Bristol Myers Squibb and Boehringer Ingelheim; lecture fees and Joint Research Fund from Bayer Healthcare; lecture fees and Scholarship Fund from Daiichi Sankyo Co., Ltd.; and grants from the Japan Agency for Medical Research and Development outside the submitted work. Dr Yamashita reported receiving lecture fees from Daiichi Sankyo Co., Ltd., Bristol Myers Squibb, Bayer, Ono Pharmaceutical, and Novartis; and advisory fees from Toa Eiyo outside the submitted work. Mr Fukuzawa and Mr Hayashi are employees of Daiichi Sankyo Co., Ltd. Dr Okumura reported receiving grants and a commission fee for the study design from Daiichi Sankyo Co., Ltd. for the submitted work and lecture fees from Daiichi Sankyo Co., Ltd., Nippon Boehringer Ingelheim, Bristol Myers Squibb, Medtronic Japan Co., Ltd., Johnson & Johnson, and Bayer Yakuhin Ltd. outside the submitted work.

## Supporting information

Figure S1–S2.

## References

[1] Lippi G , Sanchis‐Gomar F , Cervellin G . Global epidemiology of atrial fibrillation: an increasing epidemic and public health challenge. Int J Stroke. 2021;16:217–221. doi: 10.1177/1747493019897870 31955707

[2] Zathar Z , Karunatilleke A , Fawzy AM , Lip GYH . Atrial fibrillation in older people: concepts and controversies. Front Med (Lausanne). 2019;6:175. doi: 10.3389/fmed.2019.00175 31440508 PMC6694766

[3] Pisters R , Lane DA , Nieuwlaat R , de Vos CB , Crijns HJ , Lip GY . A novel user‐friendly score (HAS‐BLED) to assess 1‐year risk of major bleeding in patients with atrial fibrillation: the Euro Heart Survey. Chest. 2010;138:1093–1100. doi: 10.1378/chest.10-0134 20299623

[4] Okumura K , Akao M , Yoshida T , Kawata M , Okazaki O , Akashi S , Eshima K , Tanizawa K , Fukuzawa M , Hayashi T , et al. ELDERCARE‐AF committees and investigators. Low‐dose edoxaban in very elderly patients with atrial fibrillation. N Engl J Med. 2020;383:1735–1745. doi: 10.1056/NEJMoa2012883 32865374

[5] Kuroda M , Tamiya E , Nose T , Ogimoto A , Taura J , Imamura Y , Fukuzawa M , Hayashi T , Akao M , Yamashita T , et al. Effect of 15‐mg edoxaban on clinical outcomes in 3 age strata in older patients with atrial fibrillation a prespecified subanalysis of the ELDERCARE–AF randomized clinical trial. JAMA Cardiol. 2022;7:583–590. doi: 10.1001/jamacardio.2022.0480 35416910 PMC9008564

[6] Yoshida T , Nakamura A , Funada J , Amino M , Shimizu W , Fukuzawa M , Watanabe S , Hayashi T , Yamashita T , Okumura K , et al. Efficacy and safety of edoxaban 15 mg according to renal function in very elderly patients with atrial fibrillation: a subanalysis of the ELDERCARE‐AF trial. Circulation. 2022;145:718–720. doi: 10.1161/CIRCULATIONAHA.121.057190 35226559 PMC8876417

[7] Boriani G , Ruff CT , Kuder JF , Shi M , Lanz HJ , Antman EM , Braunwald E , Giugliano RP . Edoxaban versus warfarin in patients with atrial fibrillation at the extremes of body weight: an analysis from the ENGAGE AF‐TIMI 48 trial. Thromb Haemost. 2021;121:140–149. doi: 10.1055/s-0040-1716540 32920808

[8] Okumura K , Lip GYH , Akao M , Tanizawa K , Fukuzawa M , Abe K , Akishita M , Yamashita T . Edoxaban for the management of elderly Japanese patients with atrial fibrillation ineligible for standard oral anticoagulant therapies: rationale and design of the ELDERCARE‐AF study. Am Heart J. 2017;194:99–106. doi: 10.1016/j.ahj.2017.08.017 29223441

[9] Kadosaka T , Nagai T , Susuki S , Sakuma I , Akao M , Yamashita T , Anzai T , Okumura K . Association of low body weight with clinical outcomes in elderly atrial fibrillation patients receiving apixaban—J‐ELD AF registry subanalysis. Cardiovasc Drugs Ther. 2022;36:691–703. doi: 10.1007/s10557-021-07180-4 33830400

[10] Okumura K , Tomita H , Nakai M , Kodani E , Akao M , Suzuki S , Hayashi K , Sawano M , Goya M . Risk factors associated with ischemic stroke in Japanese patients with nonvalvular atrial fibrillation. JAMA Netw Open. 2020;3:e202881. doi: 10.1001/jamanetworkopen.2020.2881 32293685 PMC7160687

[11] Hamatani Y , Ogawa H , Uozumi R , Iguchi M , Yamashita Y , Esato M , Chun Y‐H , Tsuji H , Wada H , Hasegawa K , et al. Low body weight is associated with the incidence of stroke in atrial fibrillation patients–insight from the Fushimi AF registry. Circ J. 2015;79:1009–1017. doi: 10.1253/circj.CJ-14-1245 25740669

[12] Lee SR , Choi EK , Park CS , Han KD , Jung JH , Oh S , Lip GYH . Direct oral anticoagulants in patients with nonvalvular atrial fibrillation and low body weight. J Am Coll Cardiol. 2019;73:919–931. doi: 10.1016/j.jacc.2018.11.051 30819360

[13] Boonyawat K , Caron F , Li A , Chai‐Adisaksopha C , Lim W , Iorio A , Lopes RD , Garcia D , Crowther MA . Association of body weight with efficacy and safety outcomes in phase III randomized controlled trials of direct oral anticoagulants: a systematic review and meta‐analysis. J Thromb Haemost. 2017;15:1322–1333. doi: 10.1111/jth.13701 28407368

[14] Connolly SJ , Ezekowitz MD , Yusuf S , Eikelboom J , Oldgren J , Parekh A , Pogue J , Reilly PA , Themeles E , Varrone J , et al. RE‐LY Steering Committee and Investigators. Dabigatran versus warfarin in patients with atrial fibrillation. N Engl J Med. 2009;361:1139–1151. doi: 10.1056/NEJMoa0905561 19717844

[15] Patel MR , Mahaffey KW , Garg J , Pan G , Singer DE , Hacke W , Breithardt G , Halperin JL , Hankey GJ , Piccini JP , et al. Rivaroxaban versus warfarin in nonvalvular atrial fibrillation. N Engl J Med. 2011;365:883–891. doi: 10.1056/NEJMoa1009638 21830957

[16] Granger CB , Alexander JH , McMurray JJ , Lopes RD , Hylek EM , Hanna M , Al‐Khalidi HR , Ansell J , Atar D , Avezum A , et al. ARISTOTLE Committees and Investigators. Apixaban versus warfarin in patients with atrial fibrillation. N Engl J Med. 2011;365:981–992. doi: 10.1056/NEJMoa1107039 21870978

[17] Giugliano RP , Ruff CT , Braunwald E , Murphy SA , Wiviott SD , Halperin JL , Waldo AL , Ezekowitz MD , Weitz JI , Špinar J , et al. ENGAGE AF‐TIMI 48 Investigators. Edoxaban versus warfarin in patients with atrial fibrillation. N Engl J Med. 2013;369:2093–2104. doi: 10.1056/NEJMoa1310907 24251359

