# Supplementary material for: Efficacy and Safety of Low‐Dose Edoxaban by Body Weight in Very Elderly Patients With Atrial Fibrillation: A Subanalysis of the Randomized ELDERCARE‐AF Trial
Source: J Am Heart Assoc. 2024 Jan 19;13(3):e031506. doi: 10.1161/JAHA.123.031506 (PMC11056111; doi:10.1161/JAHA.123.031506)
Supplement: Supplementary file 1 — Figure S1–S2. [file JAH3-13-e031506-s001.pdf]

# SUPPLEMENTAL MATERIAL

**Figure S1**. **Flow chart.**

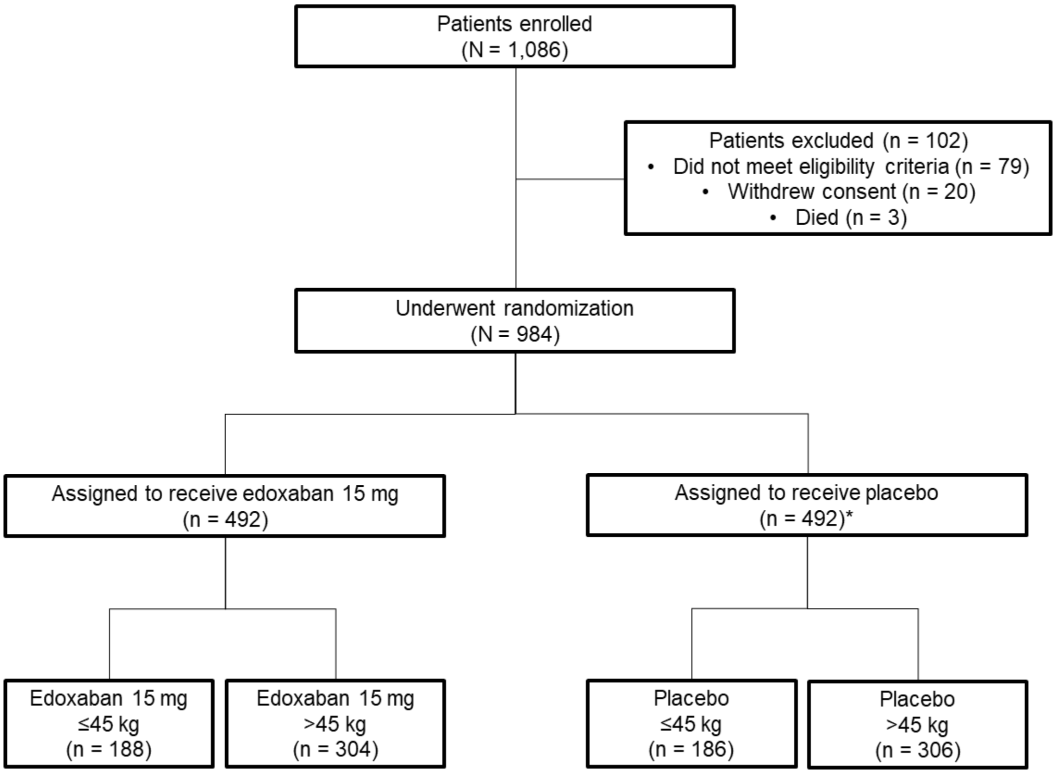


*Two patients in the placebo group withdrew from the trial before receiving the first dose and were not included in the safety population.

**Figure S2**. **Effects of edoxaban on major efficacy and safety endpoints by body weight subgroup.**

| Subgroup by Body weight | Edoxaban 15 mg | Placebo | HR (95% CI) | Interaction *P* value |
|---|---|---|---|---|
| | No. of patients with event/ Total No. of patients [Event rate % per patient-year] | | | |
| **Stroke/Systemic embolism** | | | | |
| ≤42.0 kg | 5/123 [3.3] | 13/123 [8.4] | 0.36 (0.13-1.01) | 0.64 |
| >42.0, ≤49.0 kg | 4/115 [2.6] | 15/133 [9.3] | 0.28 (0.09-0.85) | |
| >49.0, ≤57.9 kg | 2/129 [1.0] | 9/117 [5.1] | 0.20 (0.04-0.92) | |
| >57.9 kg | 4/125 [2.4] | 7/119 [4.1] | 0.62 (0.18-2.17) | |
| **All-cause Mortality** | | | | |
| ≤42.0 kg | 21/123 [13.8] | 25/123 [15.8] | 0.85 (0.48-1.53) | 0.38 |
| >42.0, ≤49.0 kg | 13/115 [8.4] | 21/133 [12.6] | 0.67 (0.34-1.34) | |
| >49.0, ≤57.9 kg | 17/129 [8.8] | 12/117 [6.7] | 1.21 (0.58-2.54) | |
| >57.9 kg | 15/125 [8.9] | 11/119 [6.4] | 1.57 (0.71-3.45) | |
| **Net Clinical Outcome** | | | | |
| ≤42.0 kg | 27/123 [18.2] | 35/123 [22.6] | 0.78 (0.47-1.29) | 0.42 |
| >42.0, ≤49.0 kg | 18/115 [12.3] | 30/133 [18.6] | 0.66 (0.37-1.18) | |
| >49.0, ≤57.9 kg | 19/129 [10.0] | 18/117 [10.3] | 0.94 (0.49-1.79) | |
| >57.9 kg | 23/125 [14.2] | 20/119 [12.0] | 1.39 (0.76-2.56) | |
| **Major Bleeding** | | | | |
| ≤42.0 kg | 6/123 [4.4] | 3/123 [2.0] | 1.97 (0.51-7.65) | 0.98 |
| >42.0, ≤49.0 kg | 6/115 [4.4] | 3/132 [2.0] | 2.20 (0.56-8.62) | |
| >49.0, ≤57.9 kg | 4/129 [2.2] | 2/116 [1.2] | 1.95 (0.31-12.27) | |
| >57.9 kg | 4/125 [2.7] | 3/119 [1.9] | 1.50 (0.39-5.78) | |
| **Major Bleeding (Gastrointestinal)** | | | | |
| ≤42.0 kg | 4/123 [2.9] | 2/123 [1.4] | 1.84 (0.34-10.05) | - |
| >42.0, ≤49.0 kg | 5/115 [3.6] | 0/132 [0.0] | - | |
| >49.0, ≤57.9 kg | 1/129 [0.6] | 1/116 [0.6] | 0.95 (0.04-20.40) | |
| >57.9 kg | 4/125 [2.7] | 2/119 [1.3] | 2.17 (0.46-10.18) | |
| **Major Bleeding or Clinically Relevant Nonmajor Bleeding** | | | | |
| ≤42.0 kg | 24/123 [18.5] | 18/123 [13.0] | 1.44 (0.79-2.65) | 0.44 |
| >42.0, ≤49.0 kg | 18/115 [14.0] | 17/132 [12.2] | 1.15 (0.60-2.22) | |
| >49.0, ≤57.9 kg | 29/129 [18.0] | 15/116 [9.6] | 1.82 (0.96-3.44) | |
| >57.9 kg | 26/125 [20.1] | 12/119 [8.2] | 2.23 (1.13-4.40) | |

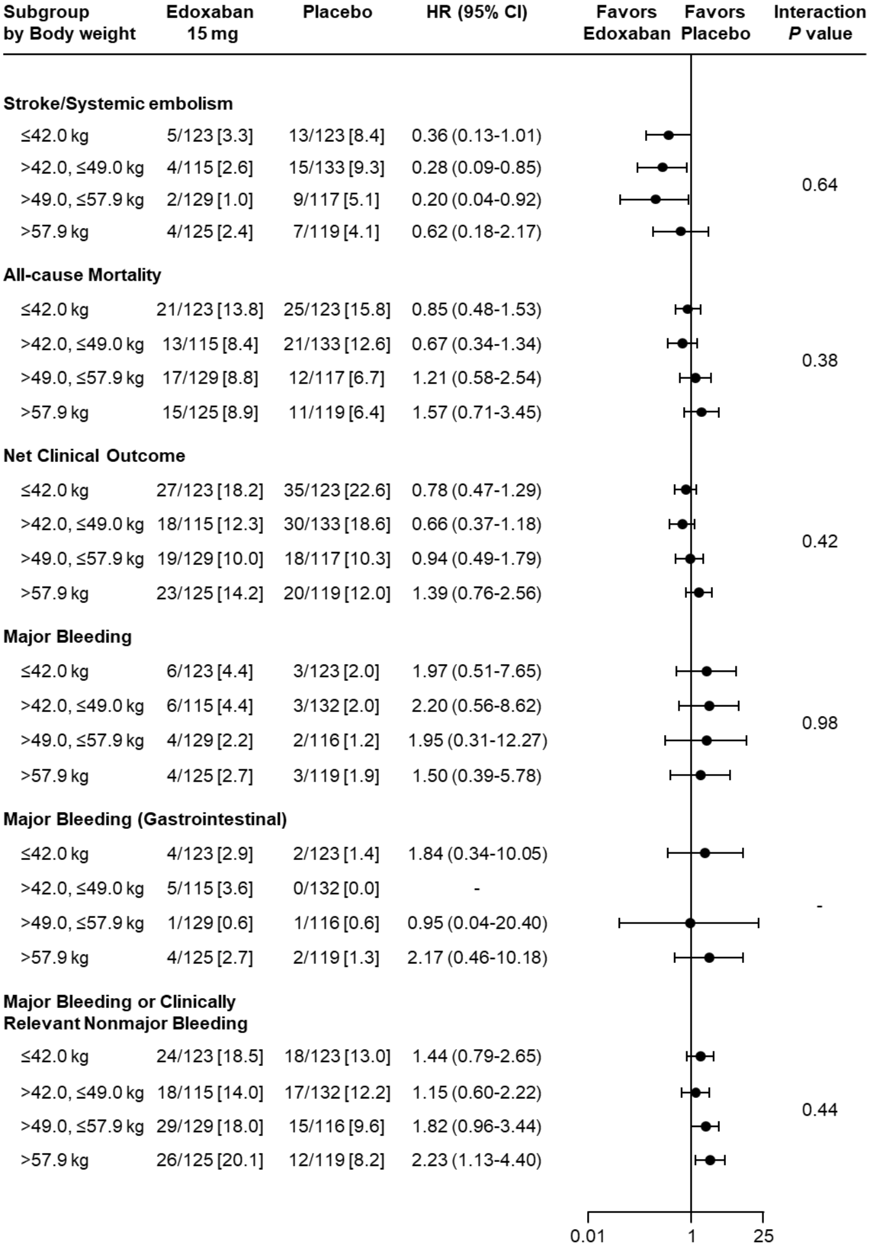


CI, confidence interval; HR, hazard ratio
